# Antibacterial activity of natural flavones against bovine mastitis pathogens: in vitro, SAR analysis, and computational study

**DOI:** 10.1007/s40203-024-00253-w

**Published:** 2024-08-24

**Authors:** Ahlam Haj Hasan, Gagan Preet, Rishi Vachaspathy Astakala, Hanan Al-Adilah, Emmanuel Tope Oluwabusola, Rainer Ebel, Marcel Jaspars

**Affiliations:** 1 Department of Chemistry, Marine Biodiscovery Centre, University of Aberdeen, Aberdeen, AB24 3UE UK; 2 Department of Medicinal Chemistry and Pharmacognosy, Faculty of Pharmacy, Jordan University of Science and Technology, Irbid, 22110 Jordan; 3 Environment and Life Sciences Research Centre, Kuwait Institute for Scientific Research, P.O. Box 24885, Safat, 13109 Kuwait

**Keywords:** Flavonoids, Bovine mastitis, In vitro, Structure-activity relationship, Molecular docking, Pharmacophore

## Abstract

Bovine mastitis is a worldwide disease affecting dairy cattle and causes major economic losses in the dairy industry. Recently, the emergence of microbial resistance to the current antibiotics complicates the treatment protocol which necessitates antibiotic stewardship and further research to find new active compounds. Recently, phytobiotics have gained interest in being used as an alternative to antibiotics in the poultry industry as an antibiotic stewardship intervention. This study evaluated the in vitro antibacterial activity of 16 flavonoids against bovine mastitis pathogens. Two flavones: 2-(4-methoxyphenyl)chromen-4-one (**1**) and 2-(3-hydroxyphenyl)chromen-4-one (**4**) showed inhibition of the growth of *Klebsiella oxytoca* with MIC values range (25–50 µg mL^− 1^) followed by a structure-activity relationship (SAR) study indicating that the presence of a hydroxyl group at C-3` or methoxy at C-4` increases the activity against *Klebsiella oxytoca* while the presence of hydroxyl group at C-7 decreases the activity. Furthermore, a structure-based drug development approach was applied using several in silico tools to understand the interactions of active flavones at the active site of the DNA gyrase protein. Compound (**4**) showed a higher docking score than quercetin (standard) which is known to have antibacterial activity by inhibiting the DNA gyrase. In addition, the structure-based pharmacophores of compound (**4**) and quercetin showed similar pharmacophoric features and interactions with DNA gyrase. Based on our findings, compounds (**1**) and (**4**) are promising for further study as potential anti-microbial phytochemicals that can have a role in controlling bovine mastitis as well as to investigate their mechanism of action further.

**Supplementary Information:**

The online version contains supplementary material available at 10.1007/s40203-024-00253-w.

## Introduction

Bovine mastitis is a worldwide disease affecting dairy cattle health and milk production and causes major economic losses in the dairy industry (Schabauer et al. 2018). Bovine mastitis is an inflammation of the bovine mammary glands caused by a defence response against bacterial infection or tissue damage. In general, the inflammatory response against harmful stimuli such as tissue damage or invading microorganisms is a normal immunological response to eliminate the harmful stimuli and start the healing process, but abnormal inflammatory response in the mammary gland may cause severe acute inflammation or chronic mastitis which negatively affects the quantity and quality of the milk and increases the mortality rate of cattle (Aitken et al. 2011; Yang et al. 2014). These consequences make bovine mastitis the most costly cattle disease (Azooz et al. 2020; Hogeveen and Van Der Voort. 2017; Hogeveen et al. 2011; Romero et al. 2018). Annual economic loss due to mastitis has been estimated to be €2 Bn in the European Union and the same figure is estimated for the USA (Wang et al. 2020). The causes of bovine mastitis are multifactorial and complex, but microbial infection is the most common cause. Several bacterial pathogens may cause bovine mastitis, and *Staphylococcus aureus* is the most common. Other common microbial causes include *Streptococcus agalactiae*, *Streptococcus dysgalactiae*, *Streptococcus uberis*, *Escherichia coli*, *Pseudomonas spp.*, and *Klebsiella spp*. (Barreiros et al. 2022; De los Santos et al. 2022; Hillerton and Berry. 2005).

Antibiotics are the recommended treatment for bovine mastitis, with b-lactams being the most used. However, several studies have reported high levels of antimicrobial resistance to antibiotics in the clinical bacterial isolates exceeding 85% such as penicillin, ampicillin, and sulphonamide in coagulase-negative *Staphylococci* (Purgato et al. 2021). Due to some pathogens` ability to resist the available antibiotics, the treatment options have become limited, and the treatment failure rate has increased (Purgato et al. 2021). The cure rate of *Staphylococcus aureus* has recently decreased due to the emergence of resistance mechanisms such as biofilm production and efflux-mediated resistance (Kamaruzzaman et al. 2017; Silva et al. 2023).

Considering the economic burden of bovine mastitis, this suggests that more productive research is needed to find new active compounds against bovine mastitis pathogens as well as antibiotic stewardship to enhance proper antibiotic use. Phytobiotics are secondary metabolites produced by plants such as flavonoids, alkaloids, and terpenes, and they are known to have several biological activities such as antimicrobial and antioxidant activities (Kikusato. 2021; Morrissey and Osbourn. 1999). Recently, phytobiotics have gained interest in being used as an alternative to antibiotics in the poultry industry as an antibiotic stewardship intervention (Abd El-Hack et al. 2016). This helps restrict the therapeutic use of antibiotics and eliminates their sub-therapeutic use (Olagaray and Bradford. 2019).

It has been reported that the presence of flavonoid in large quantities in some plant extracts such as *Schinus molle* (known as Peruvian peppertree) and *Brickellia veronicaefolia* (known as hierba dorada) leads to the inhibitory activity of these plant extracts against bovine mastitis-causing pathogens (Macías Alonso et al. 2019) as well as *Eucalyptus globulus* (known as blue gum), *Juglans regia* (known as English walnut) extracts (Gomes et al. 2019), and *Bauhinia variegate* (known as orchid tree) (Mishra et al. 2013). Flavonoids are polyphenolic natural compounds found in abundance in plants and they have essential roles in several biological processes such as growth, development, and protection against several environmental factors such as microorganisms and ultraviolet light (Shen et al. 2022). Hence, flavonoids are promising antibiotic candidates against resistant microbial pathogens (Dixon. 2001). The basic skeletal structure of flavonoids is composed of three rings and based on the degree of oxidation of the central ring, flavonoids are classified into seven subgroups (Shen et al. 2022). Flavones and flavonols are subgroups of flavonoids and distinct from other flavonoids by the presence of a double bond between C-2 and C-3 and oxidized at C-4, no substitution at C-3 position in flavone while flavonols have OH group at C-3 position (Hostetler et al. 2017; Martens and Mithöfer. 2005; Nguyen and Bhattacharya. 2022). It was found that the positions of the hydroxy and methoxy substituents in the structure play a vital role in the bioactivity of flavonoids (Chirumbolo. 2010; Preet et al. 2023). Flavones and flavonols gained much interest due to their proven bioactivity in vitro and in vivo studies (Hostetler et al. 2017; Lee et al. 1993; Martens and Mithöfer. 2005; Shen et al. 2022). A clinical study carried out on dairy cows diagnosed with mastitis, showed that the intramammary administration of micronized purified flavonoid fraction (MPFF) is effective in the treatment of bovine mastitis caused by coagulase-negative *Staphylococci* (Gutiérrez-Reinoso et al. 2023).

With the emergence of antimicrobial resistance and the increased mortality and morbidity associated with it, flavonoids have gained renewed interest as potential antimicrobial agents, especially since flavonoids are very common in plants, and have safety profiles (Amawi et al. 2017). However, their mechanism of action is still unclear (Salmanli et al. 2021). The proposed antibacterial mechanisms of flavonoids are diverse, including inhibition of nucleic acid synthesis, inhibition of energy metabolism, inhibition of biofilm formation, alteration of the permeability of cell membranes, and inhibition of the function of the cytoplasmic membrane (Xie et al. 2015; Cushnie and Lamb. 2005; Takó et al. 2020). It has been shown that flavonoids have shown a significant topoisomerase inhibition effect, leading to their antibacterial activity. For example, quercetin, apigenin, and 3,6,7,3′,4′-pentahydroxyflavone displayed a significant inhibition against DNA gyrase (topoisomerase 2), a protein responsible for DNA replication in bacteria. (Nguyen et al. 2022; Ohemeng et al. 1993; Plaper et al. 2003).

Computer-aided drug design (CADD) methods make a significant contribution to drug development research (Sadybekov and Katritch. 2023; Sliwoski et al. 2013). Structure-based methods use the information on the target and ligand structures using molecular docking, structure-based pharmacophore modelling, and molecular dynamics simulation to identify promising compounds and understand their interactions with target proteins (Palermo and De Vivo. 2015). These computational tools have significantly accelerated the drug development process (Ou-Yang et al. 2012).

In this study, the in vitro antibacterial activity of 16 flavones and flavonols against five bovine mastitis-causing pathogens was evaluated, and the minimum inhibitory concentration and the minimum bactericidal concentration were also determined. The structure-activity relationships (SAR) were analysed to determine fundamental structural properties contributing to their antibacterial activity against bovine mastitis pathogens. To understand the interactions of the tested flavonoids at the active site of the protein receptor, a molecular docking study was performed using topoisomerase II (DNA gyrase) enzyme (Bax et al. 2010; Patel et al. 2014; Shamsudin et al. 2022), and structure-based pharmacophores were generated to identify the main pharmacophoric features responsible for the antibacterial activity. Furthermore, molecular dynamics simulation was performed to assess the binding stability of the target protein with the active flavonoids.

## Methodology

### Source of compounds (1–16)

Compounds (**1**–**16**) were taken from the Marine Biodiscovery Centre Compound Library at the University of Aberdeen, UK (donated by the late Professor R.H. Thomson). The IUPAC names of these flavonoids are as follows: (**1**) 2-(4-methoxyphenyl)chromen-4-one; (**2**) 2-(3-hydroxyphenyl)-7-methoxychromen-4-one; (**3**) 7-methoxy-2-(2-methoxyphenyl)chromen-4-one; (**4**) 2-(3-hydroxyphenyl)chromen-4-one; (**5**) 8-methoxy-2-phenylchromen-4-one ; (**6**) 7-methoxy-2-phenylchromen-4-one ; (**7**) 7-hydroxy-5-methoxy-2-phenylchromen-4-one; (**8**) 7-hydroxy-2-(4-methoxyphenyl)chromen-4-one; (**9**) (4-oxo-2-phenylchromen-5-yl) acetate; (**10**) 2-(2,5-dihydroxyphenyl)-5,7-dihydroxychromen-4-one; (**11**) 2-(4-hydroxyphenyl)-3-methoxychromen-4-one; (**12**) 2-(3-ethoxy-4-methoxy-phenyl)-3,5,7-trimethoxy-chromen-4-one; (**13**) 2-(3-ethoxy-4-methoxy-phenyl)-3,5,7-trimethoxy-chromen-4-one; (**14**) 3-acetoxy-5,7-dimethoxy-2-(4-methoxy-phenyl)-chromen-4-one; (**15**) [3-acetyloxy-7-methoxy-2-(4-methoxyphenyl)-4-oxochromen-5-yl] acetate; (**16**) 2-(4-hydroxyphenyl)-3,5,7-trimethoxychromen-4-one.

### Antimicrobial assays

The antimicrobial activity of 16 flavones and flavonols was initially assessed using the Kirby-Bauer disc-diffusion assay method against five bovine mastitis strains procured from NCIMB Ltd., Aberdeen, United Kingdom. The tested pathogens are gram-positive bacteria including *Staphylococcus aureus subsp. aureus* (NCIMB 701494), *Streptococcus bovis* (NCIMB 702087), and *Enterococcus pseudoavium* (NCIMB 13084) and gram-negative bacteria included *Klebsiella oxytoca* (NCIMB 701361) and *Escherichia coli* (NCIMB 701266). Flavonoids were dissolved in dimethyl sulphoxide (DMSO), ThermoFisher Scientific Ltd., United Kingdom at a concentration of 70 µg mL^− 1^. Sterile filter paper discs were impregnated with 20 µL of flavonoid solutions and left to dry overnight. The pathogens were inoculated in Mueller-Hinton broth, Sigma Aldrich Ltd., United Kingdom for one hour in the incubator at 28 °C then spread uniformly in the form of bacterial lawn on top of Mueller-Hinton agar plate. The previously loaded discs with flavonoid solutions were placed on the inoculated plates. Sterile filter paper discs loaded with DMSO were used as the negative control and 10 µg gentamicin discs, ThermoFisher Scientific Ltd., United Kingdom as the positive control. The plates were incubated at 28 °C for 24 h and the inhibition zone was observed. The antimicrobial assay was repeated twice to confirm the results.

The minimum inhibitory concentration against the test pathogens was evaluated by the broth macro-dilution method. The broth macro-dilution method is one of the most popular antimicrobial test methods which includes preparing serial dilution (two-fold dilutions) of the tested compound in a liquid growth medium in tubes with a minimum final volume of 2 mL (Balouiri et al. 2016). The pathogens were cultured in sterile Mueller Hinton broth in test tubes with serially diluted compounds (**1**) and (**4**) in DMSO. The final concentrations of the tested compounds were: 200, 100, 50, 25,12.5, 6.25, and 3.125 µg mL^− 1^. The test tubes were placed in the incubator at 28 °C for 24 h. The minimum inhibitory concentration was determined as the lowest concentration of the compound which showed no visually observed bacterial growth (i.e., no turbidity). A test tube containing sterile Mueller-Hinton broth and another tube containing the pathogen culture were used as a negative and positive control, respectively, to confirm the media sterility and adequate microbial growth over the incubation period. An aliquot of the positive control was plated to determine the baseline concentration of the pathogens used. The minimum bactericidal concentration against the tested pathogens was performed by plating the dilution, which represents the minimum inhibitory concentration, as well as the higher concentrated dilutions on Mueller-Hinton agar plates. The minimum bactericidal concentration was determined as the lowest concentration that kills 99.9% of the final bacterial inoculum after 24 h of incubation (i.e., demonstrated no bacterial growth in the plate) (Faller et al. 2004).

### Molecular docking

**Fig. 1:**
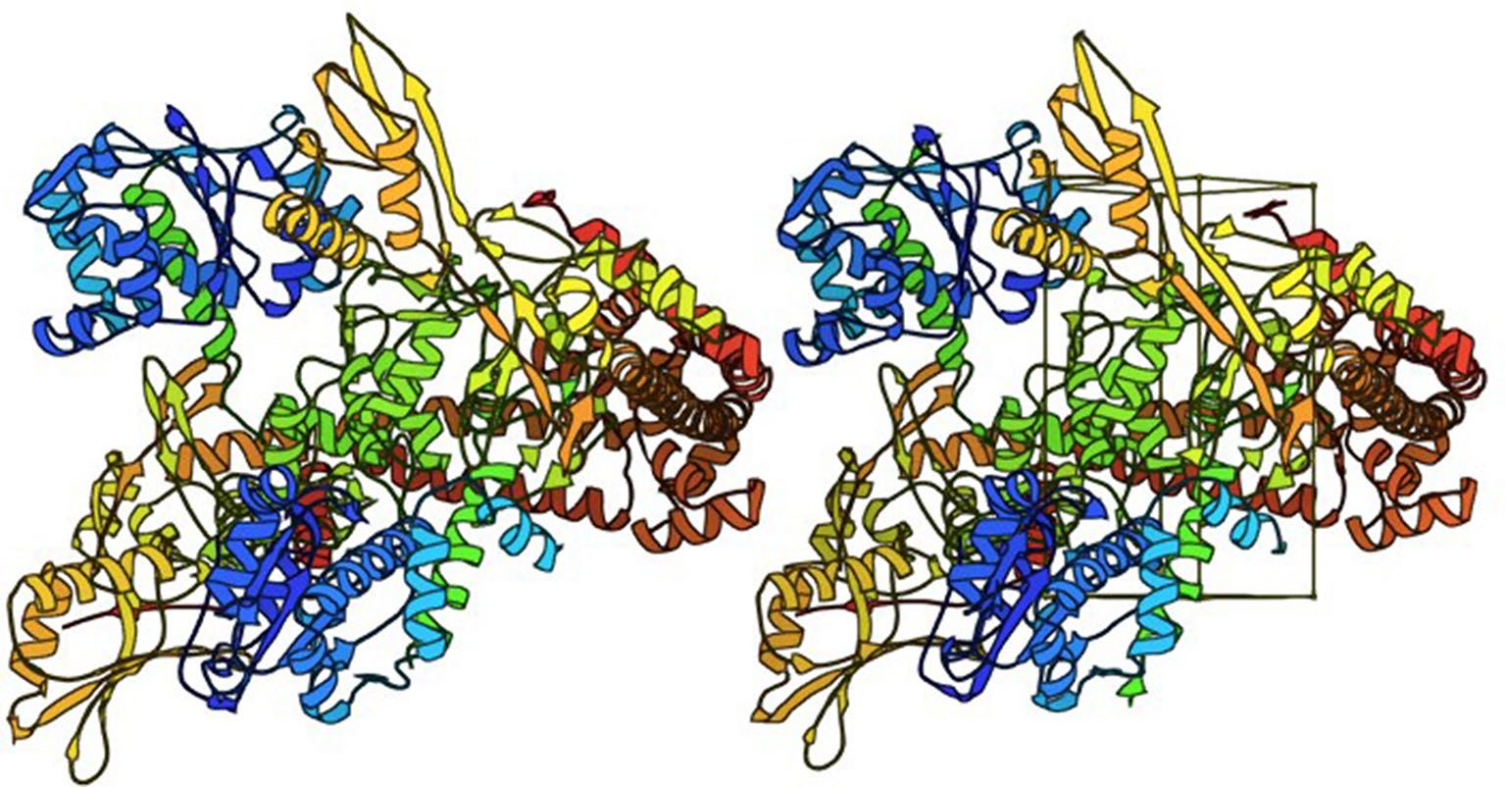
Crystal structure of DNA gyrase (PDB ID: 2XCT), the box shows the binding site

A molecular docking study was carried out using AutoDock Vina version 1.2.0. (Eberhardt et al. 2021; Trott and Olson. 2010) using Samson by OneAngstrom, 2022 (https://www.samson-connect.net/). The crystal structure of the topoisomerase II (DNA gyrase) enzyme was downloaded from the Protein Data Bank (PDB ID: 2XCT) (http://www.rcsb.org/) and the active site (i.e., interaction site) of the protein was predicted by Ligand Scout 4.4.8 software (Wolber and Thierry. 2005) which generates a box around the active site with the following box centre and size dimensions: -16.2, 43.0, 88.0 and 25.7, 52.4, 22.5 Å (Fig. 1). The binding mode number was set as 10 and the maximum energy difference was set as 3 Kcal/mol. The molecular docking was carried out using the same settings at several exhaustiveness levels for convergence. The molecular docking process involved two main steps: prediction of possible ligand conformations and their orientations inside the active site (known together as a pose) followed by evaluation of their binding energy where each pose is given a docking score. The pose which showed the highest docking score was selected in this study.

### Validation of molecular docking method

The docking method and parameters used in this study were validated by re-docking the co-crystallized ligand (Ciprofloxacin, a DNA gyrase inhibitor). This validation process involved the separation of the ligand and then re-docking the ligand again in the active site. The re-docked ligand was superimposed on the co-crystallized ligand and the Root Mean Square Deviation (RMSD) value was calculated. The low RMSD value of 0.785 Å between the experimental and the reference positions of the ligand indicated the same binding orientation which validated the method (Fig. S1).

### Structure-based pharmacophore modelling

A structure-based pharmacophore was constructed based on docked complexes of DNA gyrase protein (PDB ID: 2XCT) with compounds (**1**) and (**4**) as well as the standard flavonoid (quercetin) to further understand the interaction between the active flavonoids with the active site of the target protein at the atomic level. Docked complexes were prepared using Autodock Vina 1.2.0. (Eberhardt et al. 2021; Trott et al. 2010) which is then used to create the pharmacophores using Ligand Scout 4.4.8 software (Wolber and Thierry. 2005).

### Molecular dynamics (MD) simulation

To evaluate the conformational changes inside the active site and to assess the stability of the DNA gyrase complexes with compounds (**1**) and (**4**) over a specified time, molecular dynamics simulations were performed over 50 ns using Flare version 7.0.0 based on the OpenMM package (Eastman et al. 2017). The calculation method used was Open Force Field Explicit Water. The solvent model was Explicit TIP3P water, and the AM1-BCC method was used to compute partial charges. The following system settings were used: the solvent box shape was Truncated Octahedron, the solvent box buffer was kept at 10.0 Å, and the temperature and pressure were 298.0 K and 1.0 bar, respectively. The system was equilibrated for 200 ps. The time step 4.00 fs. The remaining settings are kept as default.

## Results and discussion

### Bioassay tests

The antimicrobial activity of **16** flavones and flavonols was tested initially using the disc diffusion assay against the following pathogens that cause bovine mastitis: *Staphylococcus aureus subsp. aureus*, *Streptococcus bovis*, *Enterococcus pseudoavium*, *Klebsiella oxytoca*, and *Escherichia coli*. The disc diffusion method is commonly used to evaluate the antimicrobial activity of pure compounds as well as microbial and plant extracts in laboratories and other institutions because of its low cost and ease of performance and interpretation (Balouiri et al. 2016). Only Compounds (**1**) and (**4**) showed a zone of inhibition at a concentration of 70 µg mL^-1^ against *Klebsiella oxytoca*. While other tested flavones and flavonols didn`t show a zone of inhibition against any of the tested pathogens. Therefore, the minimum inhibitory concentration and the minimum bactericidal concentration were determined only for compounds (**1**) and (**4**) against *Klebsiella oxytoca*. Bioassay data is summarized in (Table 1).

**Table 1:** The bioassay data of compounds (**1**) and (**4**) against *Klebsiella oxytoca*

	Zone of inhibition (mm)	Minimum inhibitory concentration (µg mL^-1^)	Minimum bactericidal concentration (µg mL^-1^)
Compound (**1**)	14 (18*)	25	200
Compound (**4**)	13 (18*)	50	200

*Zone of inhibition of positive control

### Structure-activity relationship (SAR)

Structure-Activity Relationship (SAR) is an important key to many aspects of drug discovery research, starting from the initial screening and ending up with lead optimisation (Guha. 2013). Thus, having a good understanding of the chemical properties of the flavonoids which are responsible for their bioactivity is essential in the design of new active compounds (Echeverría et al. 2017).

**Fig. 2:**
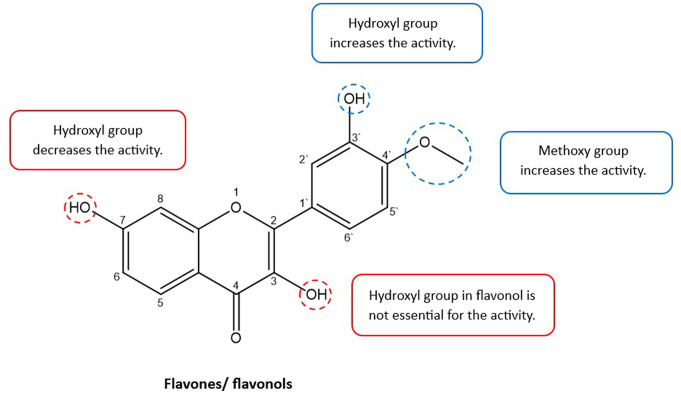
Structure-activity relationship (SAR) of the flavones and flavonols shows the possible structural features for their antibacterial activity against *Klebsiella oxytoca*

Compounds (**1**) and (**4**) showed activity against *Klebsiella oxytoca* while no flavonols showed activity against this strain. Thus, the presence of substituents at the C-3 position in the studied flavonols is not essential for the activity and might therefore decrease the antibacterial activity against this bacterial strain. It has been reported previously that hydroxylation at the C-3 position decreases antibacterial activity against gram-negative bacteria through inhibition of DNA gyrase while it is essential for antibacterial activity effect acting via the cell membrane (Shamsudin et al. 2022). Therefore, this increases the probability that the antibacterial activity of compounds (**1**) and (**4**) against *Klebsiella oxytoca* might be due to their inhibitory effect on DNA gyrase. In addition, we concluded that the presence of a hydroxyl group at C-3` or methoxy at C-4` increases the activity against *Klebsiella oxytoca* while the presence of a hydroxyl group at the C-7 position decreases the activity (Fig. 2). Although compound (**8**) has a methoxy group at C-4`, it also has a hydroxyl group at the C-7 position, which might reduce the activity.

In the literature, the antibacterial properties of flavonoids have been analysed by structure-activity relationship (SAR) studies; however, published data is inconsistent as to whether the presence of hydroxy or methoxy groups at different positions enhances or lowers the antibacterial activity. This inconsistency may be due to the amphiphilic balance (i.e., the balance between hydrophobic and hydrophilic characteristics) of the flavonoids, which is considered one of the most important aspects of antibacterial activity as it affects the ability of flavonoids to penetrate bacterial cells to exert their effect (Echeverría et al. 2017; Shamsudin et al. 2022). As mentioned above, the chemical properties of the flavonoids are essential for their antibacterial activity, however, the physical properties are also important as they affect the permeation of flavonoids through the cell wall and cell membrane of the bacteria to reach the site of action. The requirement of having phenolic groups in flavonoids for their antibacterial activity decreases the hydrophobicity and, in some cases, could affect the cell wall permeation. Hence, maintaining the balance between hydrophobicity and hydrophilicity in flavonoids is important to be taken into consideration in drug design (Echeverría et al. 2017).

### Molecular docking

**Fig. 3:**
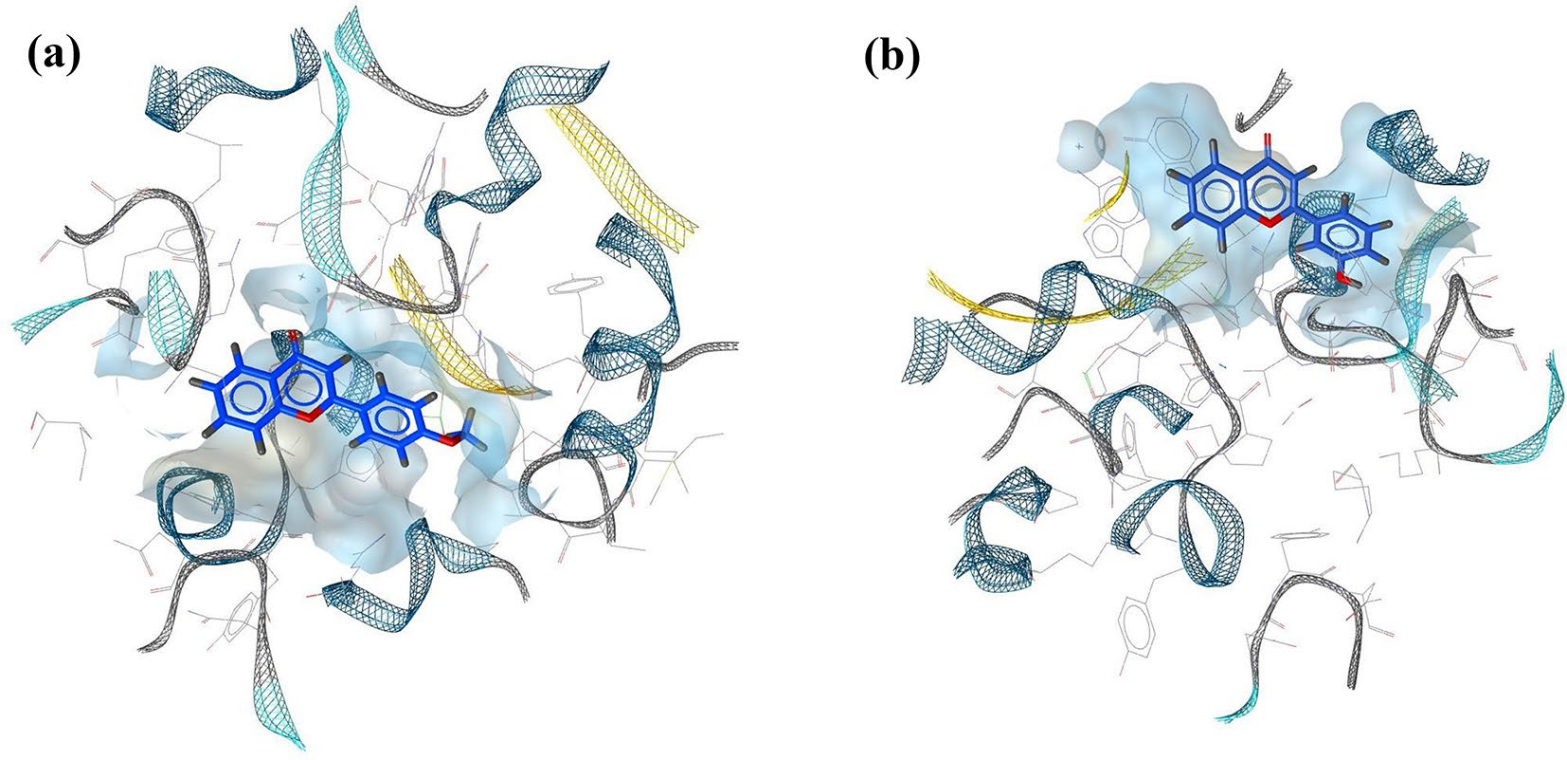
The predicted binding poses of (**a**) compound (**1**) and (**b**) compound (**4**) inside the pocket of DNA gyrase

To further understand types of interaction at the molecular level between flavonoids and the active site of the target protein, the tested flavonoids were subjected to molecular docking against the DNA gyrase enzyme which is essential for DNA replication and transcription by catalysing the ATP-dependent negative supercoiling of DNA. This enzyme is essential in all bacteria, and it is absent in human cells, thus considered an attractive target for antibiotics (Collin et al. 2011). Flavonoids are known to have antibacterial activity through their inhibitory effect on the DNA gyrase enzyme (Shamsudin et al. 2022). The tested flavonoids (**1**–**16**) were docked against DNA gyrase protein (PDB ID: 2XCT) as well as quercetin which was chosen as standard because it is one of the flavonoids that is known to exhibit antibacterial activity by binding to DNA gyrase (Hossion et al. 2011; Plaper et al. 2003). Also, ciprofloxacin was docked to validate the docking method. Generally, molecular docking uses a scoring function that ranks the ligands based on their binding energy which reflects their binding affinity to the target protein as well as proposes structural hypotheses on the interactions between ligands and the target protein (Morris and Lim-Wilby. 2008). In this study, the docking scores of quercetin and ciprofloxacin were found to be similar (-7.1 Kcal mol^− 1^). The chemical structures of the tested flavones and flavonols and docking scores are shown below (Table 2). It is interesting to note that compound (**4**) which showed antimicrobial activity against *Klebsiella oxytoca* exhibited the highest binding affinity (-7.7 Kcal mol^− 1^) to the active site of DNA gyrase protein among the tested flavones and flavonols which further increases the probability that antibacterial activity of compounds (**4**) against *Klebsiella oxytoca* might be due to its inhibitory effect on DNA gyrase protein. The predicted binding poses of compounds (**1**) and (**4**) with DNA gyrase are shown below (Fig. 3). The interactions of compounds (**1**) and (**4**) with the target protein are discussed in detail in the structure-based pharmacophore modelling section.

**Table. 2:**
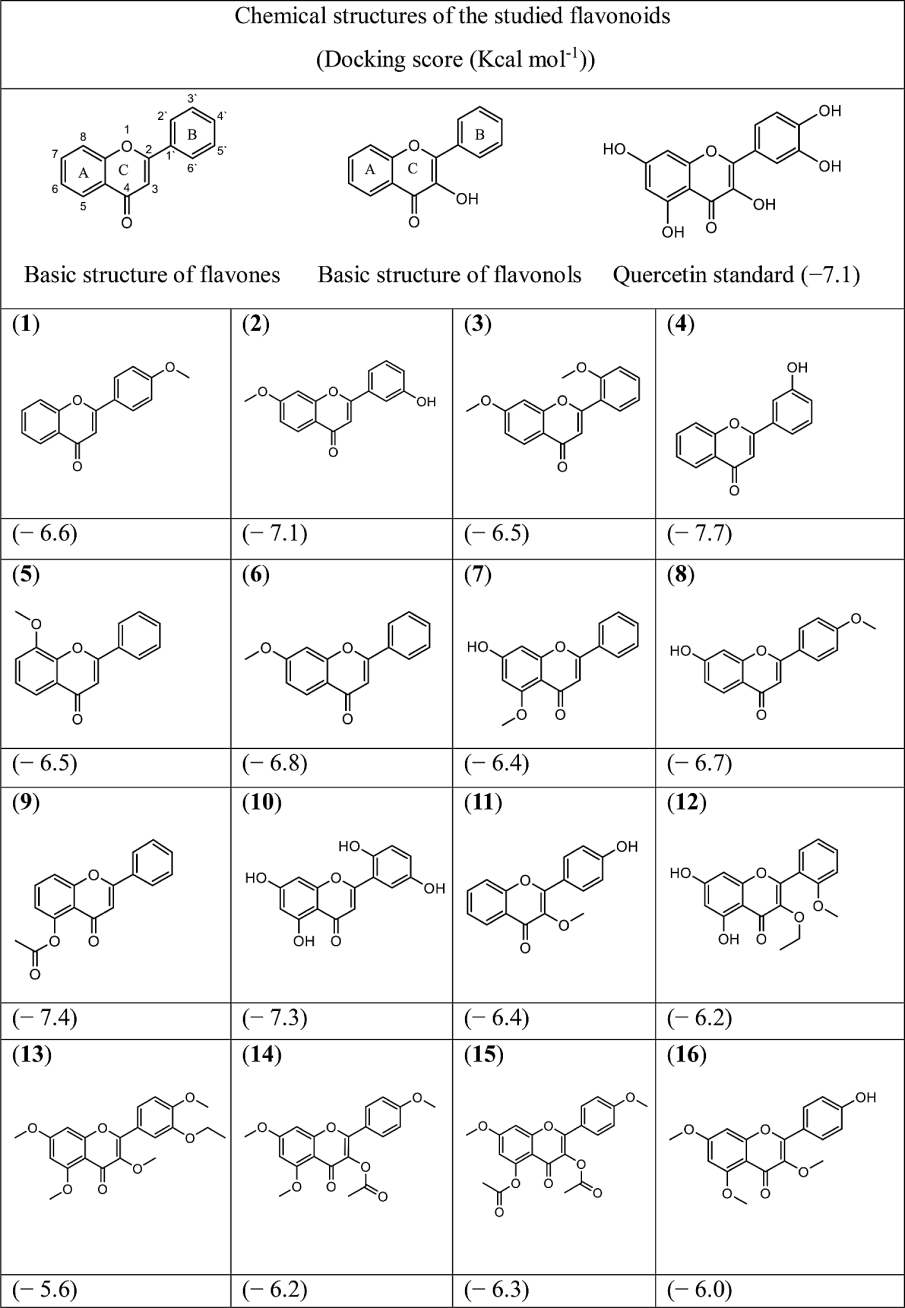
Docking scores obtained for flavonoids with DNA gyrase as the target protein by using AutoDock vina server. quercetin was used as a standard reference

### Structure-based pharmacophore modelling

**Fig. 4:**
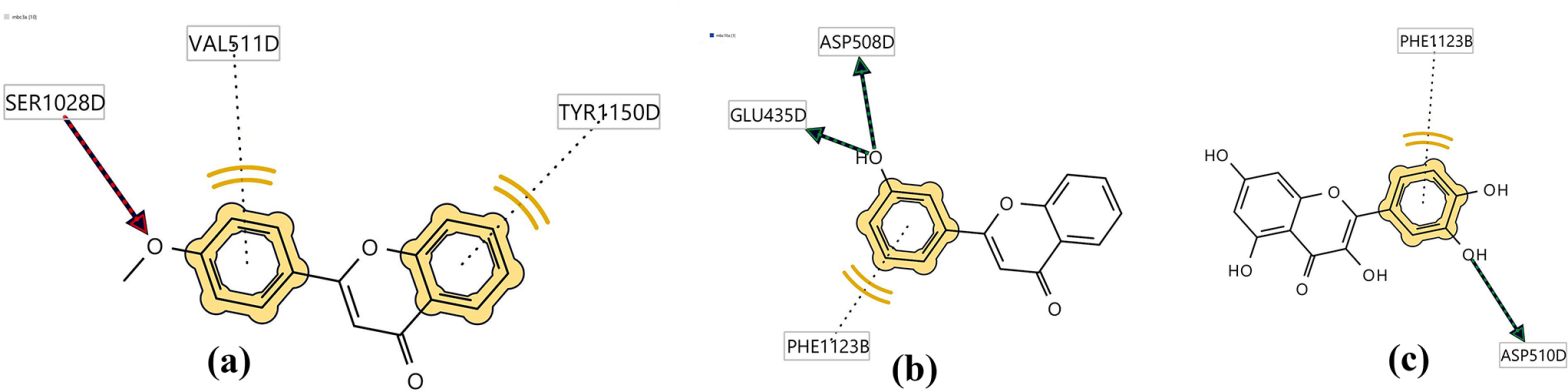
Structure-based pharmacophore of (**a**) compound (**1**), (**b**) compound (**4**), and (**c**) quercetin with DNA gyrase protein (2XCT)

Pharmacophore modelling is an important part of several computer-aided drug design (CADD) research, and it has been successfully applied in many computational methods such as virtual screening and lead optimization (Seidel et al. 2019). The definition of pharmacophore was provided by the International Union of Pure and Applied Chemistry (IUPAC) as “the ensemble of steric and electronic features that is necessary to ensure the optimal supra-molecular interactions with a specific biological target structure and to trigger (or to block) its biological response” (Wermuth et al. 1988). In this study, a structure-based pharmacophore was generated for compounds (**1**) and (**4**) using low-energy conformers where three main pharmacophoric features were identified: hydrogen bond acceptors (HBAs), hydrogen bond donors (HBDs), and hydrophobic features (H). In compound (**1**), ring A and ring B showed hydrophobic interactions with Tyr 1150D and Val 511D residues respectively, and the methoxy group at C-4` acts as a hydrogen bond acceptor for Ser1028D. In compound (**4**), Phe 1123B made a hydrophobic interaction with ring B, and the hydroxy group at C-3` acts as a hydrogen bond donor with Asp508D and Glu435D. It is worth noting that the standard (quercetin) showed the same pharmacophoric features as compound (**4**), ring B showed hydrophobic interaction with Phe 1123B and the hydroxy group at C-3` position acts as a hydrogen bond donor with Asp 510D (Fig. 4). This emphasizes the importance of Phe1123B toward the ligand-protein interaction as it interacts with compound (**4**) and quercetin. In compound (**1**), the interaction of the methoxy group at C-4` with the active site of DNA gyrase is consistent with our SAR findings about the importance of this methoxy group in the antibacterial activity of compound (**1**). Also, the interaction of the hydroxy group at C-3` in compound (**4**) with the active site of DNA gyrase confirms our SAR findings regarding the importance of this hydroxy substituent in the antibacterial activity of compound (**4**).

### Molecular dynamics (MD) simulation of DNA gyrase complexes with compounds (1) and (4)

**Fig. 5:**
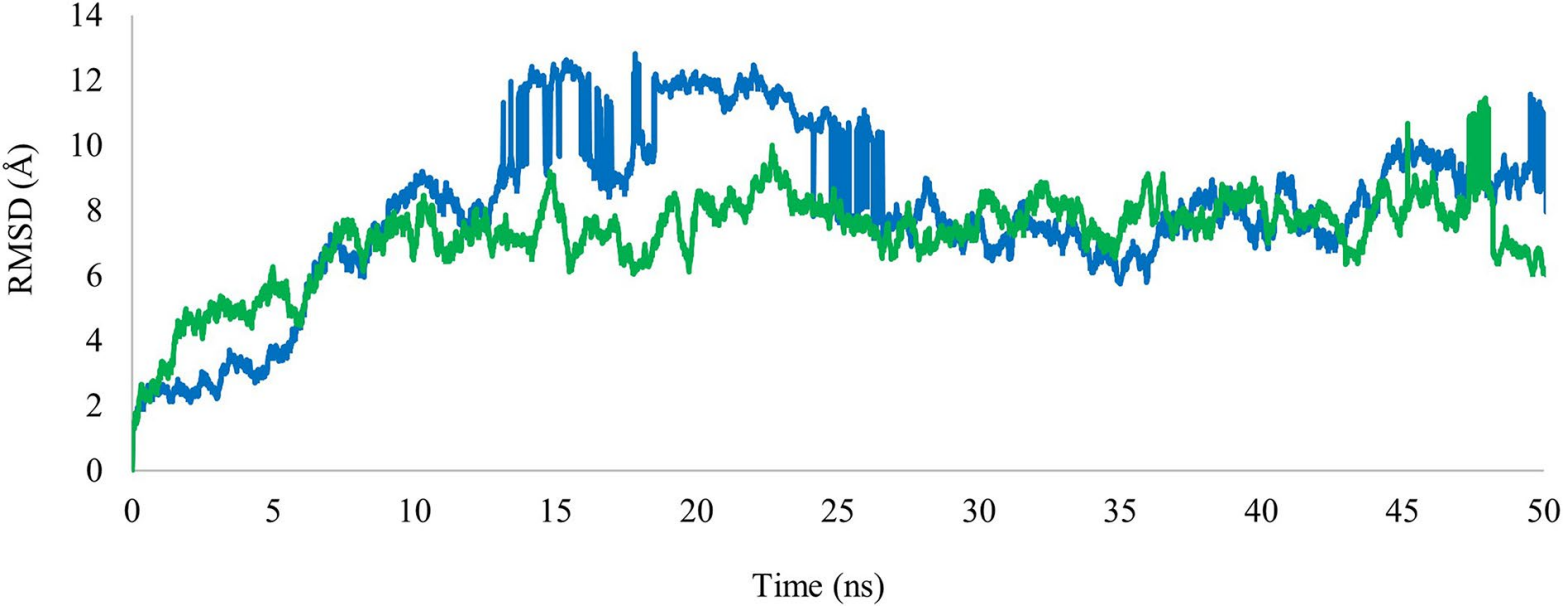
RMSD plots of DNA gyrase complexes with compound (**1**) (Blue) and compound (**4**) (Green) as a function of time

**Fig. 6:**
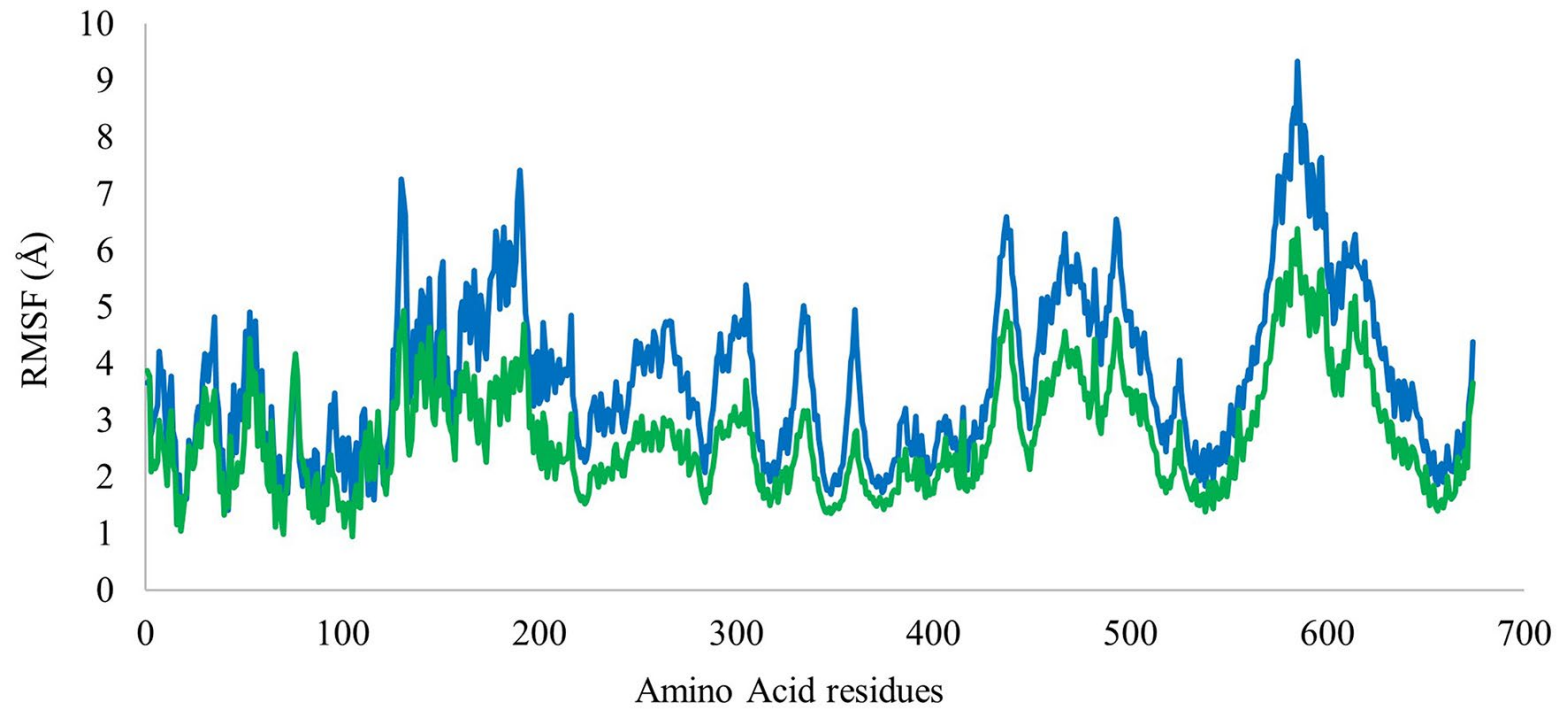
RMSF plots of compound (**1**) (Blue) and compound (**4**) (Green) in complex with DNA gyrase as a function of amino acid residue number

A molecular dynamics simulation was carried out to assess the stability of the target protein complex with ligand over a specified time and to evaluate the conformational changes inside the active site. The stability of DNA gyrase complex with compounds (**1**) and (**4**) was simulated for 50 ns using Flare version 7.0.0 using the OpenMM package version. Root Mean Square Deviation (RMSD) reflects the stability of the protein complex with the ligand over time, while the Root Mean Square Fluctuation (RMSF) reflects the flexibility of each amino acid residue of the target protein inside the complex. The RMSD of the α carbons of amino acid residues of DNA gyrase complexes with compounds (**1**) and (**4**) were found to be 8.06 ± 2.54 Å and 7.25 ± 1.36 Å respectively (Fig. 5). The large increase in RMSD values of the protein-compound (**1**) complex during the first 15 ns of the simulation reflects significant conformational changes which indicated instability. High RMSD fluctuation was also observed in the time range between 15 ns and 27 ns. The second half of the simulation showed minimal RMSD fluctuation, and the complex attained stability. RMSD values of protein-compound (**4**) complex showed an increase at the beginning of the simulation (i.e., 0–7 ns) then minimal fluctuation was observed until the end of the simulation. Therefore, the DNA gyrase complex with compound (**4**) is predicted to be relatively more stable than its complex with compound (**1**). The RMSF of DNA gyrase in the complexes with the compounds (**1**) and (**4**) were found to be 3.73 ± 1.44 Å and 2.73 ± 1.00 Å respectively (Fig. 6). Both RMSF plots were like each other which indicated that amino acids of the binding site showed similar flexibility in both complexes.

**Fig. 7:**
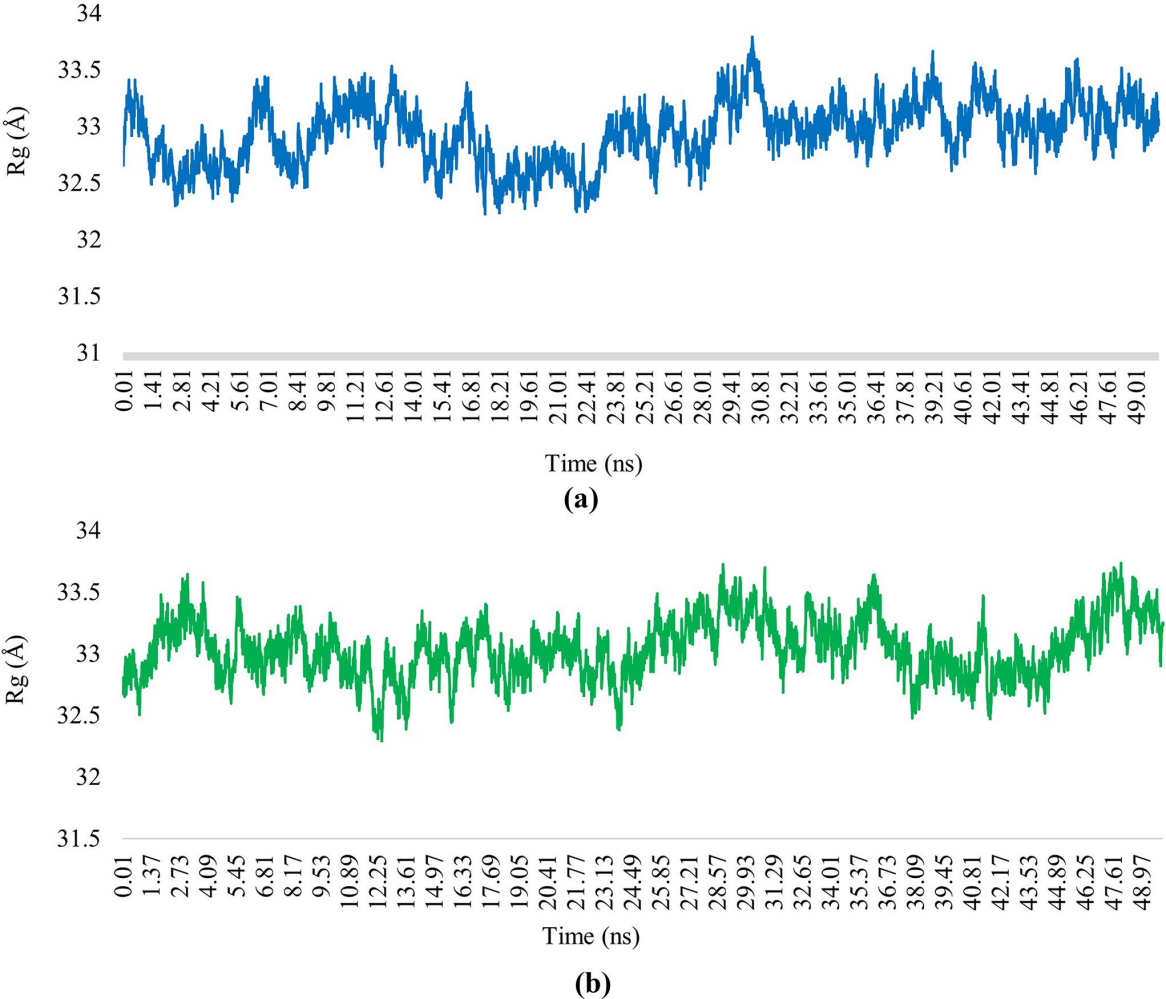
The radius of gyration (Rg) values of DNA gyrase complexes with (**a**) compound (**1**) and (**b**) compound (**4**) during 50 ns of MD simulation

The Radius of gyration (Rg) was also calculated to check the stability of the protein-ligand complex throughout the simulation time. Analysing the MD simulation of compounds (**1)** and (**4)** complexes with DNA gyrase indicated that the Rg of both fluctuated within a small range reflecting the stability of protein-ligand complexes (Fig. 7). The Rg of DNA gyrase complexes with compounds (**1**) and (**4**) were found to be 32.94 ± 0.22 Å and 33.04 ± 0.23 Å respectively.

## Conclusion

As a result of in vitro antimicrobial tests, compounds (**1**) and (**4**) showed activity against *Klebsiella oxytoca* with minimum inhibitory concentration values of 25 and 50 µg mL^− 1^, respectively. Based on the in vitro bioactivity shown by compounds (**1**) and (**4**), a structure-activity relationship (SAR) study indicates that the presence of a hydroxyl group at C-3` or methoxy at C-4` increases the activity against *Klebsiella oxytoca* while the presence of hydroxyl group at C-7 position decreases the activity. Also, a structure-based drug development approach was applied using several in silico tools including molecular docking, structure-based pharmacophore modelling, and molecular dynamics simulation. A molecular docking study showed a high affinity of these two compounds for the DNA gyrase protein, which is considered one of the target proteins that flavones act on to exhibit antibacterial activity. It is worth noting that compound (**4**) showed a higher binding affinity than the standard (quercetin) which is known to have antibacterial activity by inhibiting the DNA gyrase. In addition to that, the structure-based pharmacophores of compound (**4**) and quercetin showed similar pharmacophoric features and the same types of interactions. Based on our findings, compounds (**1**) and (**4**) are promising for further study as potential antimicrobial phytochemicals that can have a role in controlling bovine mastitis alone or in combination with other antimicrobial phytochemicals, as well as to investigate their mechanism of action further.

### Electronic supplementary material

Below is the link to the electronic supplementary material.

Supplementary Material 1

## Data Availability

No datasets were generated or analysed during the current study.
