# Supplementary material for: Antibacterial activity of natural flavones against bovine mastitis pathogens: in vitro, SAR analysis, and computational study
Source: In Silico Pharmacol. 2024 Aug 24;12(2):78. doi: 10.1007/s40203-024-00253-w (PMC11344746; doi:10.1007/s40203-024-00253-w)
Supplement: Supplementary file 1 — Supplementary Material 1 [file 40203_2024_253_MOESM1_ESM.docx]

**Supplementary Information**

**Antibacterial Activity of Natural Flavones Against Bovine Mastitis Pathogens: In Vitro, SAR Analysis, and Computational Study**

Ahlam Haj Hasan^1,2^, Gagan Preet^1^, Rishi Astakala^1^, Hanan Al-Adilah^3^, Emmanuel Oluwabusola^1^, Rainer Ebel^1^, and Marcel Jaspars^1,*^

^1^ Marine Biodiscovery Centre, Department of Chemistry, University of Aberdeen, AB24 3UE.; a.hajhasan.20@ abdn.ac.uk (AH); gagan.preet1@abdn.ac.uk (GP); r.astakala.19@abdn.ac.uk (RVA); emmanu-el.oluwabusola3@abdn.ac.uk (EO); r.ebel@abdn.ac.uk (RE); m.jaspars@abdn.ac.uk (MJ)

^2^ Department of Medicinal Chemistry and Pharmacognosy, Faculty of Pharmacy, Jordan University of Science and Technology, Irbid 22110, Jordan.

^3^ Environment and Life Sciences Research Centre, Kuwait Institute for Scientific Research, P.O. Box 24885, Safat 13109, Kuwait.; hadeelah@kisr.edu.kw (HA).

^*^ Correspondence: [m.jaspars@abdn.ac.uk](mailto:m.jaspars@abdn.ac.uk).

**Fig. S1**


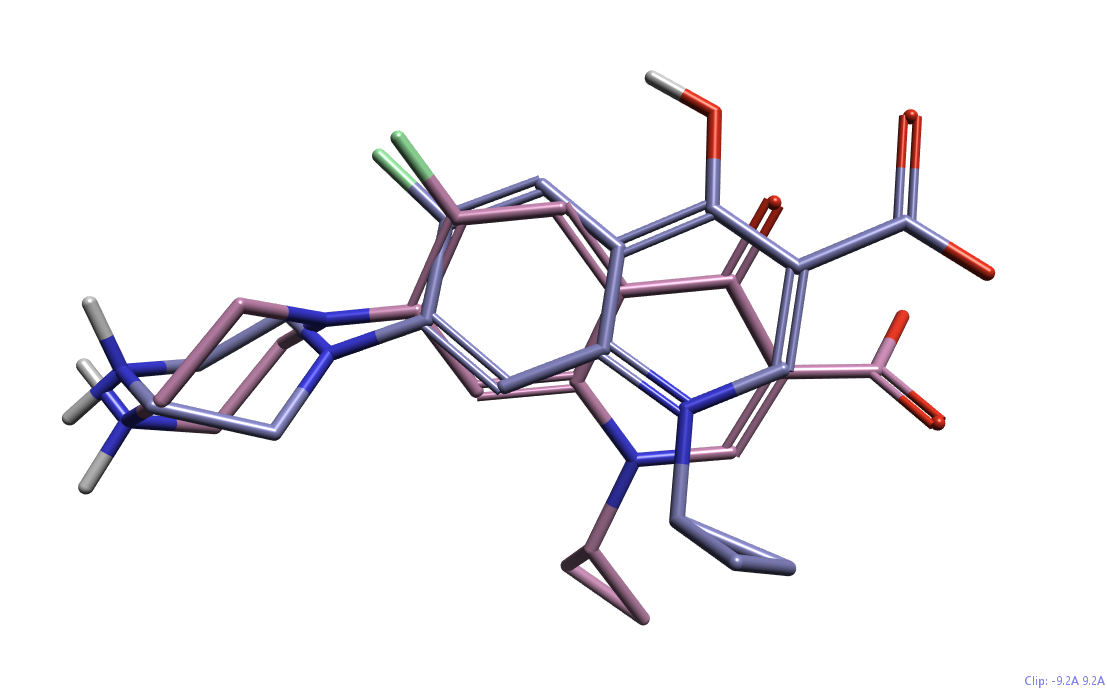


Validation of the docking method by superimposing the co-crystallized ligand (ciprofloxacin) (pink) and the re-docked ligand (gray).
